# Cytological Survey of *Malassezia* spp. Population on the Skin and External Ear Canal of Clinically Healthy Golden Retrievers and Bernese Mountain Dogs

**DOI:** 10.3390/vetsci13080733

**Published:** 2026-07-24

**Authors:** Roberta Perego, Giada Giuliani, Eva Spada, Daniela Proverbio

**Affiliations:** 1 Department of Veterinary Medicine and Animal Sciences (DIVAS), University of Milan, Via dell’Università 6, 26900 Lodi, Italy; daniela.proverbio@unimi.it; 2 Centro Vet CTO, 27029 Vigevano, Italy; giuliani.vet@gmail.com

**Keywords:** *Malassezia*, dog, cytology, skin, ear canal, Golden Retriever, Bernese Mountain Dog

## Abstract

*Malassezia* spp. are commensal yeasts of canine skin that may act as opportunistic pathogens. The aim of this study was to assess the presence, distribution and numbers of *Malassezia* spp. on the skin and external ear canal in clinically healthy Golden Retrievers and Bernese Mountain Dogs, to evaluate the influence of breed, age and sex, and to compare the findings with previously published cytological reference values. Fifty clinically healthy Golden Retrievers and 50 Bernese Mountain Dogs were included in the study. Both external ear canals and six body sites (perioral region, chin, axilla, interdigital spaces, inguinal region and dorsum) were sampled. The external ear canals were sampled using a sterile cotton swab, while the skin sites were sampled using the adhesive tape technique. The slides obtained were microscopically examined, and the number of *Malassezia* spp. was recorded. All data were statistically analyzed. *Malassezia* spp. were observed in all dogs, with different distributions in ear and skin areas. *Malassezia* spp. counts were significantly higher in Golden Retrievers than in Bernese Mountain Dogs at all skin sites. No significant difference was observed between breeds in the external ear canal. Forty-nine (98%) Golden Retrievers and 35 (74%) Bernese Mountain Dogs showed a mean skin number of *Malassezia* spp. per oil immersion field (OIF) above the 1 yeast/OIF literature threshold commonly used in the presence of clinical signs. No relationship was found between the mean value of *Malassezia* spp. per microscopic field and sex or age in either breed. The results show that Golden Retrievers have higher *Malassezia* spp. counts across all evaluated skin sites compared with Bernese Mountain Dogs. Whether higher baseline *Malassezia* spp. populations contribute to clinical yeast overgrowth requires further investigation. No significant difference in external ear canal *Malassezia* spp. counts was observed between breeds; however, the mean values recorded in both populations exceeded cytological reference values for healthy dogs. These findings indicate that cytological *Malassezia* spp. counts in clinically healthy dogs may vary according to breed and anatomical site. Diagnostic interpretation should therefore integrate cytological findings with clinical signs, breed, site, and sampling method. Further studies are required to establish normal reference intervals for *Malassezia* spp. per microscopic field based on breed and anatomical site.

## Simple Summary

*Malassezia* spp. are commensal yeasts that form part of the normal cutaneous and auricular microbiota of dogs, but which can act as opportunistic pathogens in several dermatological conditions. In clinical practice, cytological evaluation is widely used to assess yeast overgrowth; however, there is still no clear consensus on normal reference values, and currently used thresholds do not account for potential variability between breeds and anatomical sites. In this study, we evaluated the presence, distribution, and number of skin and external ear canal *Malassezia* spp. in clinically healthy Golden Retrievers and Bernese Mountain Dogs. Our results show that Golden Retrievers have significantly higher baseline yeast populations compared to Bernese Mountain Dogs across multiple skin sites. In addition, *Malassezia* spp. numbers in a substantial proportion of clinically healthy dogs from both breeds exceeded previously published cytological skin and ear *Malassezia* spp. reference values. These findings suggest that commonly used cytological thresholds may require cautious interpretation and may not be equally applicable across all breeds and anatomical sites. These findings may help refine the clinical interpretation of cytological findings in canine dermatology.

## 1. Introduction

*Malassezia* spp. are lipophilic, non-mycelial, unipolar budding yeasts forming part of the cutaneous microbiota of many warm-blooded animals, including dogs [1,2]. The non-lipid-dependent *Malassezia pachydermatis* is considered the most common commensal yeast isolated from the skin, mucosal surfaces, and external ear canal of healthy dogs [1,2,3,4,5,6].

Although typically commensal, *Malassezia* yeasts are recognized as opportunistic pathogens and are frequently involved in the pathogenesis of otitis externa and superficial dermatitis [1,3]. The transition from commensalism to pathogenicity is influenced by multiple host- and environment-related factors, including genetic predisposition, hypersensitivity disorders, keratinization abnormalities, endocrine diseases, and alterations in the cutaneous microenvironment [2,3]. *Malassezia* dermatitis and otitis, inflammatory conditions associated with increased populations of *M. pachydermatis*, are being reported with increasing frequency in dogs [3].

Despite their clinical relevance, the definition of normal *Malassezia* populations in healthy dogs remains controversial [3]. Cytological evaluation is widely used in clinical practice; however, only a limited number of studies have attempted cytological quantification of *Malassezia* spp. on the skin [4,6,7] and in the external ear canal [8,9,10] of healthy dogs, and there is no universal agreement regarding the number of yeasts that can be considered to be “within physiological limits”. Furthermore, *Malassezia* spp. numbers appear to vary considerably between different anatomical sites, contributing to variability in diagnostic interpretation [4,6,11]. Chen and Hill [3] suggest, in view of the conflicting data, that finding at least one *Malassezia* spp. per oil-immersion microscopic field (OIF) in the presence of clinical signs can usually be taken to indicate a skin overgrowth of *M*. *pachydermatis* in dogs. Breed-related differences in *Malassezia* populations have also been suggested. Previous studies in healthy dogs have reported higher yeast numbers in some breeds such as Beagles and Basset Hounds compared to mixed-breed dogs [5,12].

In ear canal cytology, there is also no definite amount of yeast that is considered clinically normal, with a mean of 0.2 [11] to <2 [8,9,10] yeast on high-power magnification microscopic field (HPF), and studies are absent in different canine breeds. This missing data is important, as more information would help to clarify the pathogenic role of *Malassezia* spp. in skin and auricular diseases [3,13].

The Golden Retriever is a widely distributed large-breed dog throughout Europe and the United States. Originally developed as a hunting and retrieving breed, it is predisposed to several dermatological conditions [14,15], which may influence the cutaneous microenvironment and could potentially be associated with higher baseline *Malassezia* spp. populations. In contrast, no specific predisposition to *Malassezia*-associated diseases has been reported in Bernese Mountain Dogs, a large Swiss farm breed originally used for guarding, draught work, and cattle driving. Both breeds are large-sized dogs with double coats, reducing potential confounding related to body size and coat type. For these reasons, the Bernese Mountain Dog was considered a suitable comparator breed in the present study.

To the authors’ knowledge, no cytological studies have investigated the carriage of *Malassezia* spp. on the skin and in the external ear canal of clinically healthy dogs belonging to these two breeds. Therefore, the aim of the present study was to evaluate and compare the presence, distribution, and population size of *Malassezia* spp. on the skin and in the external ear canal of clinically healthy Golden Retrievers and Bernese Mountain Dogs, and to assess the potential influence of breed, age, and sex. Additionally, the results were compared with cytological reference values reported in the literature. We hypothesized that clinically healthy Golden Retrievers would show higher baseline cutaneous *Malassezia* spp. counts than Bernese Mountain Dogs and that yeast counts would vary according to anatomical site.

## 2. Materials and Methods

### 2.1. Study Population

Clinically healthy Golden Retrievers and Bernese Mountain Dogs from ten breeding kennels located in Northern Italy were enrolled in a study spanning an entire calendar year and covering all four seasons. Kennels were selected according to breeder availability, and each kennel housed only one breed. Approximately ten dogs were enrolled from each kennel. All dogs were enrolled during routine health checks with the owners’ informed consent and with approval from the Animal Welfare Committee of the University of Milan (OPBA_102_2018).

The enrolled subjects had no history of skin or ear disease and were deemed healthy based on history, physical examination, and results of a CBC and basic biochemical analysis.

On the day of sampling, a dermatological examination was performed to confirm the absence of signs of dermatological disease such as coat greasiness, alopecia, acanthosis, lichenification, erythema, scaling, papules/pustules, ulcers or crusts. The absence of pruritus was evaluated using a previously validated owner-reported pruritus severity scale [16,17]. Dogs with signs consistent with skin disease or with pruritus were excluded.

The external ear canal was otoscopically evaluated to exclude signs of otitis externa, including erythema, edema, scaling, crusting, alopecia, exudate, and evidence of discomfort or pain. Additionally, signs suggestive of otitis externa, such as head shaking and scratching, were assessed using the same pruritus scale previously mentioned. Dogs with signs consistent with otitis externa were excluded.

None of the dogs had received topical (including shampoo therapy) or systemic antifungal, antibiotic, or immunosuppressive treatments in the three months preceding sampling. In addition, none of the dogs had been bathed within the two weeks preceding sampling. No female was in oestrus, pregnant, or lactating at the time of sampling, and none of the dogs were receiving fatty acid supplementation, probiotics, or other topical dermatological products.

For each dog, a clinical record was completed including signalment data (breed, sex, and age) and owner information.

### 2.2. Cytological Sampling

Cytological samples were collected from six different skin sites selected based on the previous literature regarding the distribution of *Malassezia* spp. on canine skin [3,4,5,6,12,18]. These sites included: right and left perioral region (sites B1 and B2), chin (site C), right and left axilla (sites D1 and D2), interdigital region (site E), inguinal region (site F), and dorsum (site G). Samples from the chin, inguinal region, dorsum, and interdigital region were obtained by sampling randomly selected areas or randomly selected interdigital spaces.

Skin samples were collected using the Scotch^®^ tape-strip technique. A 5 cm strip of adhesive tape was applied by the same operator to the skin with gentle pressure and then removed firmly. This procedure was repeated with the same strip of adhesive tape three times for each site [19]. The collected samples were stained using a modified Wright’s rapid stain (Quick Panoptic kit; Pokler, Salerno, Italia), mounted with mineral oil and covered with a coverslip for microscopic evaluation.

For each dog, cytological samples from the external ear canal were collected using two sterile cotton swabs (one per ear, sites A1 and A2). Each swab was inserted into the ear canal and gently rotated at the junction between the vertical and horizontal canal. The collected material was rolled onto a clean microscope slide to create a thin, even smear. Slides were heat-fixed, stained using a modified Wright’s stain (Quick Panoptic kit; Pokler, Salerno, Italy), mounted with mineral oil, and covered with a coverslip, as previously described [20].

### 2.3. Microscopic Analysis

Slides were evaluated by one trained observer who was blinded to breed. Skin slides were initially examined at low magnification (100×) to identify representative areas, then at high-power magnification (400×) to detect microorganisms morphologically consistent with *Malassezia* spp., and, finally, under oil immersion (1000×) for quantitative evaluation. At 1000× magnification, ten non-overlapping randomly selected microscopic fields were examined for each slide after excluding areas with staining artefacts, tape folds, or excessively thick preparations that could interfere with microscopic evaluation. The number of *Malassezia* spp. observed in each field was recorded, and the mean number of yeasts per oil immersion field (OIF) was subsequently calculated for each slide.

Ear cytology slides were also first examined at low magnification (100×) and then at high-power magnification (400×). At this magnification, ten randomly selected microscopic fields were examined for each slide after excluding areas with staining artefacts or excessively thick preparations. The number of *Malassezia* spp. observed in each field was recorded, and the mean number of yeasts per high-power field (HPF) was subsequently calculated for each slide. The HPF magnification was chosen for ear samples to allow comparison with previously published studies [8,9,10].

### 2.4. Statistical Analysis

All statistical analyses were performed using commercial software (MedCalc, version 12.3.0; MedCalc Software bvba, Mariakerke, Belgium).

Possible differences in demographic characteristics between the two study populations were assessed prior to data analysis. The distribution of sex between Golden Retrievers and Bernese Mountain Dogs was compared using a chi-square test, while mean age was compared using an unpaired *t*-test. The D’Agostino–Pearson test was used to assess the normality of data distribution within each group (Golden Retriever and Bernese Mountain Dog). For statistical analysis, bilateral samples (A1 and A2, B1 and B2, D1 and D2) were averaged to obtain one representative value for each anatomical site (A, B and D) for each dog [21]. Subsequently, the mean *Malassezia* spp. count across the six sampled skin sites (B–G) was calculated for each dog and used for the comparison of overall skin yeast populations between breeds and for the evaluation of the effects of age and sex. Thus, the dog and the site, rather than the individual slide, were considered the main biological units for breed comparisons. Descriptive statistics were calculated for each sampled site and reported as mean ± standard deviation (SD), median (interquartile range, IQR), minimum, and maximum values. Depending on data distribution, either an unpaired Student’s *t*-test or a Mann–Whitney U test was used to evaluate differences in mean *Malassezia* spp. counts between sampled sites within each breed, as well as between the two breeds. No formal correction for multiple comparisons was applied; therefore, individual site-level comparisons were interpreted cautiously, with emphasis placed on the consistency and magnitude of the observed differences across anatomical sites. The mean number of *Malassezia* spp. per field (OIF for skin and HPF for ear samples) was compared with the skin cytological reference value of 1 yeast/OIF [3], as well as with published reference values/HPF for ear cytology (Table 1). The association between age and total *Malassezia* spp. skin and ear counts was assessed using Spearman’s rank correlation coefficient, whereas differences in total *Malassezia* spp. skin and ear counts between male and female dogs were evaluated using the Mann–Whitney U test. Missing or non-evaluable samples were excluded only from the analysis of the corresponding anatomical site. No data imputation was performed.

Statistical significance was set at *p* < 0.05 for all analyses.

## 3. Results

### 3.1. Study Population

The study included a total of 100 purebred dogs. The Golden Retriever (GR) group consisted of 50 dogs: 24 intact males (48%) and 26 females (52%) (24 intact and two spayed), with an age range of 6 months to 14 years (mean ± SD: 4.9 ± 3.92 years). The Bernese Mountain Dog (BMD) group also included 50 dogs: 14 males (28%) (13 intact and one neutered) and 36 females (72%) (33 intact and three spayed), with an age range of 6 months to 11 years (mean ± SD: 3.1 ± 2.81 years). Although the sex distribution differed between breeds, the difference did not reach statistical significance (*p* = 0.064). In contrast, Golden Retrievers were significantly older than Bernese Mountain Dogs (*p* = 0.006).

### 3.2. Cytological Sampling and Microscopic Evaluation

For technical reasons (damaged or inadequately stained samples), complete evaluation of all sites was not possible in a small number of cases; in the GR group, 49/50 dogs had complete sampling of all study sites, while 1/50 dogs had incomplete sampling (6/7 sites sampled, axilla site missing). In the BMD group, 4/50 dogs had incomplete sampling: three dogs had only ear samples available, while one dog only lacked evaluation of the axilla site.

Additionally, ear canal data from eight dogs (five from the GR group and three from the BMD group) were excluded from statistical analysis due to the presence of inflammatory cells and phagocytosed bacteria on slides, which is consistent with cytological evidence of bacterial otitis, despite the absence of clinical findings or symptoms. Including these ear canal data in the statistical analysis did not materially change the comparison between breeds; therefore, only the predefined analysis excluding ears with cytological evidence of bacterial otitis is presented. These dogs were retained in the study and included in all skin analyses, as no cutaneous abnormalities were detected.

The final number of cytological slides included in the statistical analysis for each anatomical site is reported in Table 2.

### 3.3. Prevalence of Malassezia spp.

At the dog level, *Malassezia* spp. yeasts were observed in all 100 dogs (100%) included in the study, with at least one positive sampled site in each dog.

At the slide level, *Malassezia* spp. were detected in all analyzed slides from the Golden Retriever group (488/488; 100%) and in 438 out of 468 (93.6%) analyzed slides from the Bernese Mountain Dogs. In the remaining 30 slides, no yeasts were observed. These negative samples were distributed across different anatomical sites (10 perioral, three chin, six axilla, four interdigital space, four inguinal, and three dorsum) in different subjects.

### 3.4. Cytological Findings

In the GR group, the mean number of *Malassezia* spp. on the skin (excluding the external ear canal site) evaluated in 50/50 dogs was 2.3 ± 0.8 yeasts/OIF. The minimum observed skin value was 0.2 yeasts/OIF (dorsum site), while the maximum observed skin value was 8.3 yeasts/OIF (perioral site). The mean number of *Malassezia* spp. in the ear canal evaluated in 45/50 dogs was 2.8 ± 1.9 yeasts/HPF. The minimum number observed in the ear was 0.6 yeasts/HPF, while the maximum observed ear value was 9.7 yeasts/HPF.

In the BMD group, the mean number of *Malassezia* spp. on the skin (excluding the external ear canal site) evaluated in 47/50 dogs was 1.4 ± 0.7 yeasts/OIF. The minimum observed skin value was 0 yeasts/OIF for each skin site, while the maximum observed skin value was 4.9 yeasts/OIF (perioral site). The mean number of *Malassezia* spp. on the ear canal evaluated in 47/50 dogs was 3.2 ± 4.2 yeasts/HPF. The minimum observed number in the ear was 0.1 yeasts/HPF, while the maximum was 22.4 yeasts/HPF.

**Figure 1:**
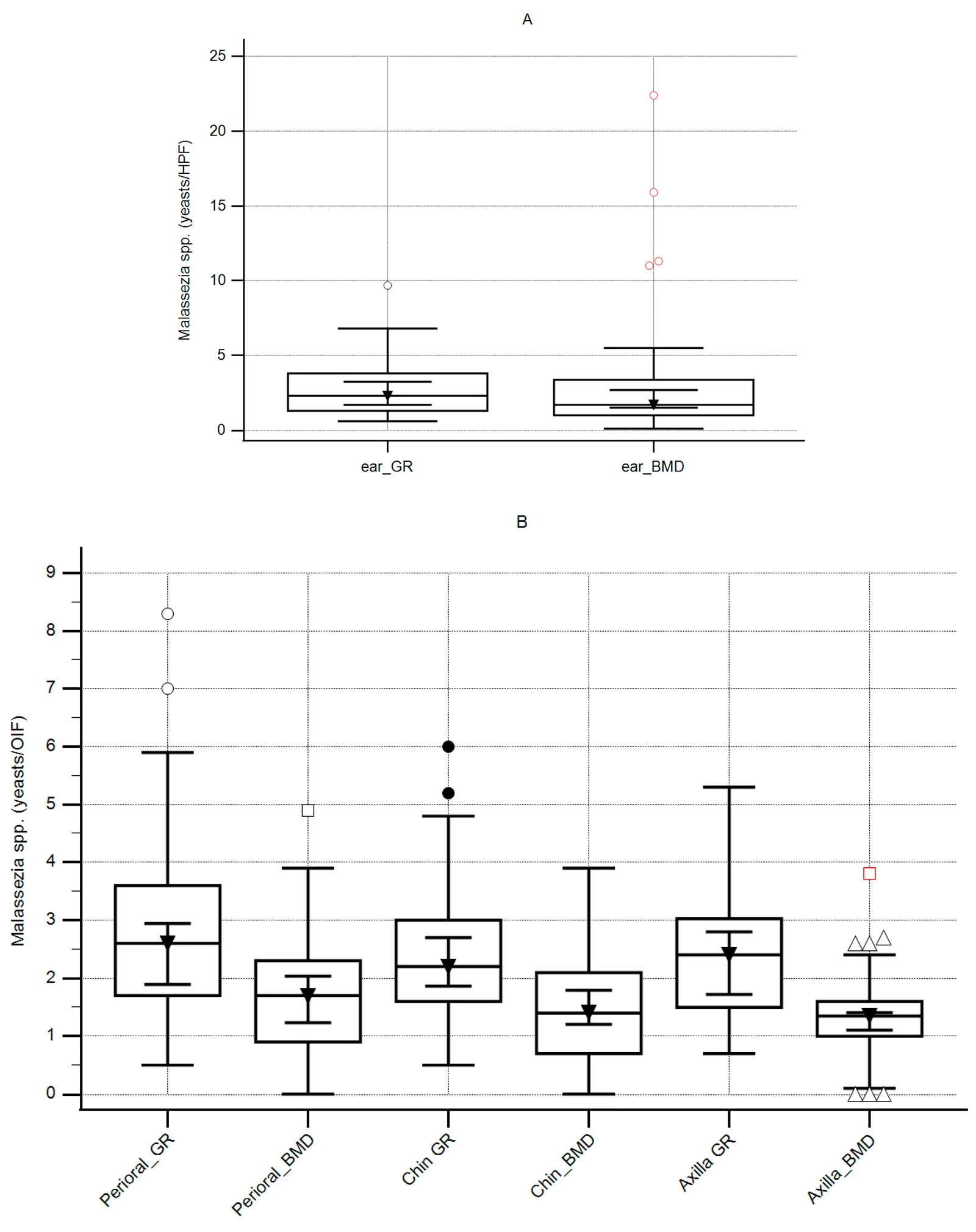

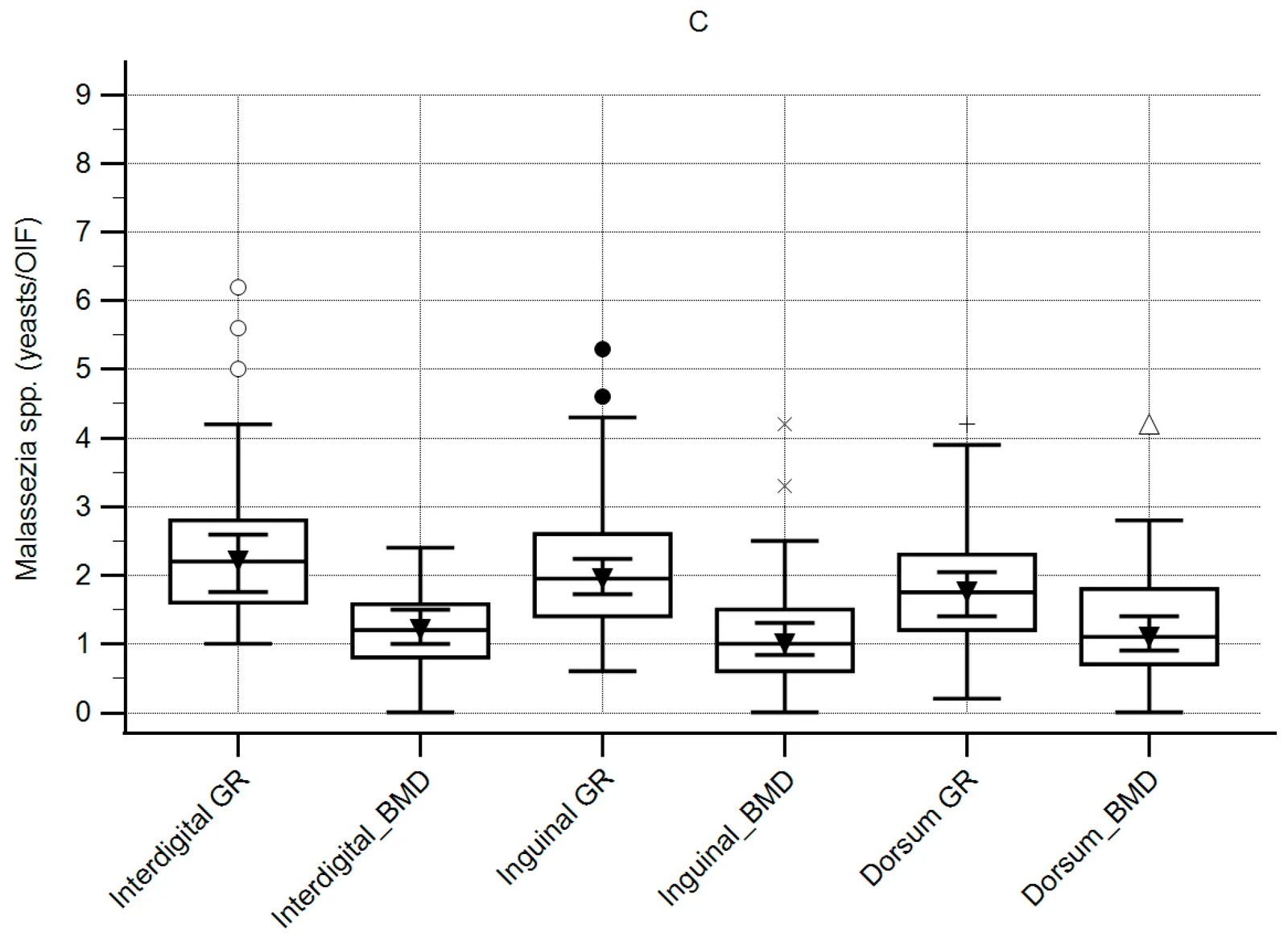
Box-and-whisker plots show the distribution of *Malassezia* spp. counts in Golden Retrievers (GRs) and Bernese Mountain Dogs (BMDs). (**A**) External ear canal (yeasts/HPF). (**B**) Perioral region, chin and axilla (yeasts/OIF). (**C**) Interdigital space, inguinal region and dorsum (yeasts/OIF). The central line represents the median, the box represents the interquartile range (IQR), the whiskers represent the range excluding outliers, and circles indicate outliers.

Detailed minimum, median, mean, and maximum values for each anatomical site are reported in Table 3 and Figure 1.

### 3.5. Comparison Between Skin Sites

In the GR group, the mean number of *Malassezia* spp. on the dorsum (site G) was significantly lower compared to the perioral region (site B), chin (site C), axilla (site D), and interdigital space (site E) (Table 3). The mean number of *Malassezia* spp. on the perioral region (site B) was significantly higher compared to the inguinal region (site F). No significant differences were observed between the remaining sites.

In the BMD group, the perioral region (site B) showed significantly higher *Malassezia* spp. numbers compared to the interdigital space (site E), inguinal region (site F), and dorsum (site G). The chin (site C) also showed significantly higher numbers compared to the inguinal region (site F). No other significant differences were observed among the remaining sites (Table 3).

### 3.6. Comparison Between Breeds

The overall mean skin population of *Malassezia* spp. (excluding the external ear canal) was significantly higher in Golden Retrievers compared to Bernese Mountain Dogs (*p* < 0.0001). Furthermore, for each individual skin site, the mean yeast count was significantly higher in the GR group compared to the BMD group. No significant difference was observed between breeds in the external ear canal (*p* = 0.2861) (Table 3).

### 3.7. Comparison with Literature Reference Values

In both groups, skin *Malassezia* spp. counts frequently exceeded the reported cytological reference value of 1 yeast/OIF reported in the literature in the presence of clinical signs. Specifically, 49 out of 50 Golden Retrievers (98%) and 35 out of 47 Bernese Mountain Dogs (74%) showed a mean number of yeast above this threshold.

For the external ear canal, the mean number of yeast observed in Golden Retrievers (2.8 yeasts/HPF) and Bernese Mountain Dogs (3.2 yeasts/HPF) exceeded all previously reported reference values [8,9,10] (Table 1).

### 3.8. Influence of Age and Sex

No statistically significant association was found between total skin and external ear canal counts of *Malassezia* spp. and age (respectively rho = −0.11, *p* = 0.45 and rho = −0.02, *p* = 0.87 for Golden Retrievers; rho = −0.11, *p* = 0.46 and rho = −0.13, *p* = 0.38 for Bernese Mountain Dogs). No significant difference was observed between male and female dogs in total skin *Malassezia* spp. counts (*p* = 0.70 for Golden Retrievers and *p* = 0.40 for Bernese Mountain Dogs) and external ear canal *Malassezia* spp. counts (*p* = 0.10 for Golden Retrievers and *p* = 0.62 for Bernese Mountain Dogs).

## 4. Discussion

To the authors’ knowledge, this is the first large-sample-size epidemiological cytological study evaluating the population of *Malassezia* spp. on the skin and in the external ear canal of two clinically healthy purebred populations of Golden Retrievers and Bernese Mountain Dogs.

The results confirm that *Malassezia* spp. are part of the normal cutaneous and auricular microflora of clinically healthy dogs, as previously reported [4,5]. *Malassezia* spp. were detected in all 100 dogs included in the study and in nearly all cytological samples, being present in 100% of slides from the Golden Retriever group and 93.6% of slides from the Bernese Mountain Dog group.

Previous studies have shown that the distribution of *Malassezia* spp. on canine skin is not uniform across different anatomical sites [4,5,6]. In agreement with these findings, the present study demonstrated significant variations in yeast numbers depending on the body region. In Golden Retrievers, the dorsum region showed significantly lower yeast numbers compared to other sites, and the perioral region showed significantly higher yeast numbers compared to the inguinal region. In Bernese Mountain Dogs, the perioral and chin regions were among the most densely colonized areas, while fewer Malassezia spp. numbers were seen in the interdigital, inguinal, and dorsal regions.

These findings are consistent with previous studies. Cafarchia et al. (2005) [6] reported that the perioral region is one of the most densely colonized areas for *Malassezia* spp., whereas lower numbers are seen in the inguinal region. Kennis et al. (1996) [4] found greater yeast numbers in the chin compared to axillary and inguinal regions. Bond et al. (1995) [5] also reported higher numbers in the perioral region compared to other sites.

Minor and partial differences between the present study and previous reports may be explained by variations in sampling techniques (impression using glass slides, cotton swabs or skin scraping versus tape strip technique), as already proposed by Cafarchia (2005) [6]. Further variations may be explained by differences in study populations and in the number of animals included in each study. While previous studies often involved small and heterogeneous populations, the present study evaluated a relatively large number of clinically healthy dogs belonging to only two breeds, which may provide more consistent data.

A key finding of this study is the significantly higher skin numbers of *Malassezia* spp. observed in Golden Retrievers compared to Bernese Mountain Dogs. This difference was evident across all skin sites and when considering overall skin yeast counts.

This finding may be partly explained by breed-specific characteristics. Golden Retrievers possess a dense, water-resistant double coat with a thick undercoat and were originally developed as water-retrieving dogs. These coat characteristics may contribute to a skin microenvironment that favours the persistence and proliferation of *Malassezia* spp. In addition, this breed is predisposed to dermatological conditions such as atopic dermatitis and ichthyosis [22,23], which may further contribute to increased *Malassezia* spp colonization, even in clinically healthy individuals. To the authors’ knowledge, the higher baseline *Malassezia* spp. counts observed in clinically healthy Golden Retrievers have not been previously reported in the literature. Whether these higher baseline *Malassezia* populations contribute to an increased risk of clinical *Malassezia* dermatitis remains unknown and cannot be determined from the present cross-sectional study. Longitudinal studies will be required to clarify whether dogs with higher baseline yeast populations are more likely to develop clinical disease in the presence of additional predisposing factors.

Although certain breeds, such as West Highland White Terriers, Poodles, and Cocker Spaniels [24], are considered to be predisposed to *Malassezia* dermatitis, quantitative studies comparing yeast populations between breeds are limited. The only studies looking at the population of *Malassezia* spp. in specific canine breeds are on purebred Beagles and Basset Hounds [5,12,18,25,26]. Bond et al. (1995) [5] compared the population of *Malassezia* spp. at skin and mucosal sites in a group of 20 clinically healthy Beagles with a group of 20 healthy dogs of different breeds and reported larger numbers of *Malassezia* spp. in Beagles. Bond et al. (1997) [12] compared 13 healthy Basset Hounds with 20 healthy mixed-breed dogs and reported larger numbers of *Malassezia* spp. in Basset Hounds. This was confirmed by Bensignor et al. (2002) [18] in a study of 20 Basset Hounds with samples taken from four areas of the body (auricular pinna, axillary region, umbilical region and perianal region).

Another important finding in this study is that a large proportion of clinically healthy dogs from both breeds had mean skin yeast counts exceeding the cytological threshold of 1 yeast/OIF [3]. This was observed in 98% of Golden Retrievers and 74% of Bernese Mountain Dogs. The mean yeast count on the skin of Golden Retrievers was found to be twice (2.30 yeast/OIF) the cytological threshold, whereas in Bernese Mountain Dogs it was only slightly higher (1.35 yeast/OIF).

Although the findings in Golden Retrievers may partly reflect breed-specific characteristics, their interpretation remains challenging. One possible explanation is the considerable variability in the literature regarding normal *Malassezia* spp. counts in healthy dogs, which led Chen and Hill [3] to propose a theoretical threshold of 1 yeast/OIF in the presence of clinical signs. However, it should be noted that the dogs included in the present study were clinically healthy and showed no dermatological signs suggestive of *Malassezia* dermatitis. In addition, to the authors’ knowledge, no epidemiological studies specifically evaluating baseline *Malassezia* spp. populations in clinically healthy Golden Retrievers are available for comparison. The finding of *Malassezia* spp. numbers slightly above the theoretical limit in the Bernese Mountain Dog (a breed chosen as comparator), however, also requires further investigation. However, it should be noted that, in this breed, the mean was elevated by counts from the perioral and chin sites. These anatomical sites are generally characterized by increased moisture due to their proximity to the oral cavity and have previously been reported as areas of higher *Malassezia* colonization [6]. The comparison with the 1 yeast/OIF value should therefore be regarded as exploratory rather than diagnostic, as this threshold was originally proposed to assist the interpretation of cytological findings in dogs presenting compatible clinical signs rather than in clinically healthy individuals. Accordingly, cytological counts should always be interpreted together with clinical findings, lesion distribution, inflammatory cytology, underlying allergic, endocrine or keratinization disorders, response to antifungal therapy and other diagnostic information, rather than as an isolated numerical criterion for the diagnosis of *Malassezia* dermatitis. A high cytological count in a clinically healthy dog should not be considered equivalent to *Malassezia* dermatitis.

With regard to the external ear canal, no significant difference in *Malassezia* spp. counts was observed between Golden Retrievers and Bernese Mountain Dogs (*p* = 0.2861). Although the mean ear canal count was slightly higher in Bernese Mountain Dogs, this reflected considerable variability within the group, as indicated by the large standard deviation and the wide range of observed values, with only a small number of dogs showing markedly higher counts than the remainder of the population. The mean ear canal yeast counts recorded in both breeds exceeded the cytological reference values currently reported for clinically healthy dogs [8,9,10]. However, comparisons with previously published ear cytology reference values should be interpreted with caution, as differences in sampling technique, staining methods, microscopic magnification, counting procedures, and study populations may influence the reported yeast counts. Taken together, these findings highlight an important limitation of currently used cytological reference values, which are largely based on heterogeneous canine populations and may not be universally applicable across breeds or anatomical sites. The results of the present study suggest that breed- and site-specific reference values may improve the interpretation of cytological findings in clinical practice. However, the present findings should be regarded as preliminary evidence of breed- and site-associated variation in cytological *Malassezia* spp. counts rather than as definitive breed- or site-specific reference values. Larger studies including broader dog populations and multivariable analyses are required before robust breed- and site-specific reference values can be established. No significant association was found between yeast population and sex or age in either group. Although the proportion of females was higher in the Bernese Mountain Dog group, no statistically significant association between sex and *Malassezia* spp. counts was detected within either breed, suggesting that sex was unlikely to have substantially influenced the observed breed differences, as previously reported [2,27]. Regarding age, the Golden Retriever population was significantly older than the Bernese Mountain Dog population. Although no association between age and *Malassezia* counts was detected within either breed, the possibility of residual confounding related to age cannot be completely excluded and should be addressed in future studies. Nevertheless, recruiting clinically healthy dogs of similar age from different pure breeds is challenging and represents a common limitation of observational studies of this type. Some studies have reported higher *Malassezia* spp. colonization in puppies than in older dogs [27]. This association was not observed in the present study; however, dogs younger than 6 months of age were not included, and the number of dogs under 1 year of age was limited, potentially reducing our ability to detect age-related differences.

In our study, external ear canal data from eight dogs (five from Golden Retrievers and three from Bernese Mountain Dogs) were excluded from the statistical analysis because, although at the time of sampling the subjects did not have signs suggestive of otitis externa, bacterial otitis (inflammatory cells and phagocytosed bacteria) was detected cytologically. This finding highlights the value of routine cytological evaluation of the external ear canal, as subclinical inflammatory or infectious changes may be present even in the absence of overt clinical signs [10].

Some limitations of the present study should be acknowledged. First, the present study was designed as a cytological survey because cytology represents the diagnostic technique most commonly used in routine veterinary dermatology. Consequently, fungal culture was not included in the study design, and it was therefore not possible to identify the different *Malassezia* species present. Although *M. pachydermatis* is considered the predominant *Malassezia* species in dogs [6], cytological examination alone does not allow species-level identification. Therefore, the present findings refer to *Malassezia* spp. rather than to a specific species. Other *Malassezia* species have also been reported in dogs and may have different pathogenic roles [6,27,28]. Future investigations combining cytology with fungal culture and/or molecular identification would help determine whether the breed-related differences observed in the present study are also associated with differences in species composition.

Second, environmental factors such as housing conditions and seasonality were not specifically analyzed. As many of the enrolled dogs were show dogs, housing conditions and grooming practices (i.e., frequent bathing associated with participation in dog shows) may have differed from those of the general pet population. These factors could potentially influence cutaneous colonization by *Malassezia* spp. and may limit the generalizability of the findings. Also, seasonality may influence the cutaneous microenvironment and, consequently, yeast populations [3,24,29]. It is appropriate, however, to emphasize that sampling in our study was done throughout one year, including all the different seasons and weather conditions, which should have mitigated this bias. Although some enrolled dogs may have been related, pedigree relationships were not systematically recorded and therefore could not be evaluated. Likewise, kennel was not included as an analytical variable because the study was not designed to investigate kennel-level effects. Furthermore, other potentially relevant variables, including kennel management, diet, environmental humidity, and genetic relatedness among dogs, were not systematically recorded and therefore could not be evaluated. These factors may also contribute to variation in cutaneous *Malassezia* populations and should be considered in additional prospective studies. Additionally, the dogs were considered clinically healthy based on their medical history, clinical examination, and routine blood tests, but no hormonal evaluation (e.g., assessment of thyroid function) was performed, and this may have limited the detection of subclinical endocrinological conditions.

Videotoscopy was not used to facilitate the sampling of the material at the level of the junction between the horizontal and the vertical portion of the external ear canal. This was not considered to be indicated as the subjects were clinically healthy but may have limited the standardization of the sampling procedure. However, previous studies on ear cytology in healthy subjects [8,9] have used a similar method of investigation.

A further limitation of the present study is that repeated measurements within the same dog and potential clustering by kennel or season were not modelled using mixed-effects approaches. Although bilateral samples were averaged before statistical analysis and the observed breed differences were consistent across all evaluated skin sites, further studies using multivariable mixed-effects models would be useful to account for within-dog correlation and additional potential sources of clustering.

Finally, the tape-strip technique was performed without prior clipping of the hair coat [18,19], as many enrolled dogs were show dogs and clipping was not acceptable to the owners. Consequently, tape adherence to the skin surface may have been reduced, potentially leading to a slight underestimation of cutaneous *Malassezia* spp. counts.

## 5. Conclusions

To the authors’ knowledge, this study is the first to report significantly higher *Malassezia* spp. populations in the skin of clinically healthy Golden Retrievers compared with clinically healthy Bernese Mountain Dogs. No significant difference in external ear canal *Malassezia* spp. counts was observed between breeds; however, the mean values recorded in both populations exceeded cytological reference values previously reported for healthy dogs.

Given the opportunistic pathogenic role of these yeasts and the importance of quantitative cytological evaluation in veterinary dermatology, these findings highlight the need to consider breed-related differences when interpreting cytological results. Furthermore, the observed variability in yeast populations across anatomical sites supports the need for more precise, site-specific reference values.

Future studies should aim to establish breed- and site-specific cytological reference intervals and to further investigate the role of environmental and host-related factors in *Malassezia* colonization. In addition, studies incorporating fungal culture and/or molecular identification may provide further insight into breed-related differences in *Malassezia* populations.

## Figures and Tables

**Table 1:** Cytological reference values for *Malassezia* spp. in the external ear canal reported in the literature (HPF, 400× magnification).

Author	Study Population	Reference *Malassezia* NV
Ginel et al., 2002 [8]	37 healthy dogs (different breeds)	<2 yeasts/HPF
Tater et al., 2003 [9]	50 healthy dogs (different breeds)	<0.2 yeasts/HPF
Cafarchia et al., 2005 [10]	82 healthy dogs (crossbreeds)	<2 yeasts/HPF

NV: normal value.

**Table 2:** Number of dogs and cytological slides included in the statistical analysis for each anatomical site in Golden Retriever and Bernese Mountain Dog groups. Differences in skin slide numbers reflect missing or non-evaluable samples due to technical issues and the exclusion of ear samples showing cytological evidence of bacterial otitis.

Site	Anatomical Location	GR Dogs Sampled	GR Slides Analyzed	BMDs Sampled	BMD Slides Analyzed	Slides Excluded
A	External ear canal (A1+A2)	45/50	90	47/50	94	5 GR + 3 BMD (bacterial external otitis)
B	Perioral region (B1+B2)	50/50	100	47/50	94	3 BMD (technical reasons
C	Chin	50/50	50	47/50	47	3 BMD (technical reasons
D	Axilla (D1+D2)	49/50	98	46/50	92	1 GR + 4 BMD (technical reasons)
E	Interdigital space	50/50	50	47/50	47	3 BMD (technical reasons
F	Inguinal region	50/50	50	47/50	47	3 BMD (technical reasons
G	Dorsum	50/50	50	47/50	47	3 BMD (technical reasons
	Total number of slides analyzed		488		468	

A1 = right ear, A2 = left ear, B1 = right perioral region, B2 = left perioral region, D1 = right axilla, D2 = left axilla, GR = Golden Retriever, and BMD = Bernese Mountain Dog.

**Table 3:** Minimum (Min), median (interquartile range, IQR), mean ± standard deviation (SD), and maximum (Max) number of *Malassezia* spp. observed by optical microscopy in Golden Retriever (GR) and Bernese Mountain Dog (BMD) in different sites, examining 10 randomly selected fields. The *p*-value column reports the comparison between GR and BMD groups for each anatomical site. Bold characters and asterisks indicate anatomical sites involved in significant (*p* < 0.05) within-breed pairwise comparisons, as described in the text.

Site	Location	Golden Retriever				Bernese Mountain Dog				*p*-Value
		Min	Median (IQR)	Mean ± SD	Max	Min	Median (IQR)	Mean ± SD	Max	
**A**	External ear canal (yeast/HPF)	0.6	2.3 (2.5)	2.82 ± 1.94	9.7	0.1	1.7 (2.3)	3.20 ± 4.18	22.4	0.2861
**B**	Perioral (yeast/OIF)	0.5	2.6 (1.9)	**2.80 ± 1.58** *	8.3	0	1.7 (1.4)	1.69 ± 1.10 *	4.9	0.0001
**C**	Chin (yeast/OIF)	0.5	2.2 (1.4)	2.46 ± 1.21 *	6	0	1.4 (1.4)	1.51 ± 0.94 *	3.9	<0.0001
**D**	Axilla (yeast/OIF)	0.7	2.4 (1.5)	2.33 ± 0.99 *	5.3	0	1.35 (0.6)	1.32 ± 0.77	3.8	<0.0001
**E**	Interdigital (yeast/OIF)	1	2.2 (1.2)	2.42 ± 1.11 *	6.2	0	1.2 (0.7)	1.20 ± 0.66 *	2.4	<0.0001
**F**	Inguinal (yeast/OIF)	0.6	1.95 (1.2)	2.15 ± 1.05 *	5.3	0	1.0 (0.9)	1.16 ± 0.84 *	4.2	<0.0001
**G**	Dorsum (yeast/OIF)	0.2	1.75 (1.1)	1.85 ± 0.95 *	4.2	0	1.1 (1.1)	1.21 ± 0.85 *	4.2	0.0007
	Mean of skin sites B-G (yeast/OIF)	0.6	2.3 (1)	2.30 ± 0.78	5.9	0	1.4 (0.9)	1.35 ± 0.66	3.9	<0.0001

Min = minimum number, Max = maximum number, HPF = high-power field, OIF = oil immersion field, and IQR = interquartile range.

## Data Availability

The original contributions presented in this study are included in the article. Further inquiries can be directed to the corresponding authors.
